# The Function and Organization of Lateral Prefrontal Cortex: A Test of Competing Hypotheses

**DOI:** 10.1371/journal.pone.0030284

**Published:** 2012-02-15

**Authors:** Jeremy R. Reynolds, Randall C. O'Reilly, Jonathan D. Cohen, Todd S. Braver

**Affiliations:** 1 Department of Psychology, University of Denver, Denver, Colorado, United States of America; 2 Department of Psychology, University of Colorado Boulder, Boulder, Colorado, United States of America; 3 Department of Psychology and Princeton Neuroscience Institute, Princeton University, Princeton, New Jersey, United States of America; 4 Department of Psychology, Washington University, Saint Louis, Missouri, United States of America; University College London, United Kingdom

## Abstract

The present experiment tested three hypotheses regarding the function and organization of lateral prefrontal cortex (PFC). The first account (the *information cascade hypothesis*) suggests that the anterior-posterior organization of lateral PFC is based on the timing with which cue stimuli reduce uncertainty in the action selection process. The second account (the *levels-of-abstraction hypothesis*) suggests that the anterior-posterior organization of lateral PFC is based on the degree of abstraction of the task goals. The current study began by investigating these two hypotheses, and identified several areas of lateral PFC that were predicted to be active by both the information cascade and levels-of-abstraction accounts. However, the pattern of activation across experimental conditions was inconsistent with both theoretical accounts. Specifically, an anterior area of mid-dorsolateral PFC exhibited sensitivity to experimental conditions that, according to both accounts, should have selectively engaged only posterior areas of PFC. We therefore investigated a third possible account (the *adaptive context maintenance hypothesis*) that postulates that both posterior and anterior regions of PFC are reliably engaged in task conditions requiring active maintenance of contextual information, with the temporal dynamics of activity in these regions flexibly tracking the duration of maintenance demands. Activity patterns in lateral PFC were consistent with this third hypothesis: regions across lateral PFC exhibited transient activation when contextual information had to be updated and maintained in a trial-by-trial manner, but sustained activation when contextual information had to be maintained over a series of trials. These findings prompt a reconceptualization of current views regarding the anterior-posterior organization of lateral PFC, but do support other findings regarding the active maintenance role of lateral PFC in sequential working memory paradigms.

## Introduction

During the past decade, considerable attention has been given to understanding the processes associated with various areas of lateral prefrontal cortex (PFC). This work has consistently suggested that areas of lateral PFC are organized in some fashion along an anterior-posterior gradient [1]–[7]. However, despite apparent agreement on the presence of such a gradient, there is substantial controversy regarding the representational and/or processing demands that underlie this organization. Two strongly formulated hypotheses have received considerable attention:

1. The *information cascade hypothesis:* The anterior-posterior gradient of lateral PFC is organized according to when cue stimuli reduce uncertainty in (i.e. provide information useful for) the action selection process [4], [5], [8]. Anterior areas of lateral PFC are postulated to respond selectively to task cues that are temporally remote from the action selection process, and therefore must be maintained for extended durations (i.e., across multiple trials). In contrast, posterior areas are postulated to be responsive to cues that appear in close temporal proximity to the action selection process (i.e., in the same trial) in addition to cues that are relevant across trials. Because this hypothesis relies upon information theory to quantify the cascading contributions of multiple control signals, we subsequently refer to it as the *information cascade hypothesis*.

2. The *levels-of-abstraction hypothesis:* The anterior-posterior gradient of PFC is organized according to the level of abstraction (or hierarchical nesting) of cues required to guide action selection [1], [9]. Anterior areas of lateral PFC are associated selectively with the processing of more abstract information regarding actions (e.g. sets of stimulus-response mappings), whereas posterior areas of PFC are postulated to be associated with the processing of more concrete information regarding actions (e.g. individual stimulus-response mappings).

**Figure 1:**
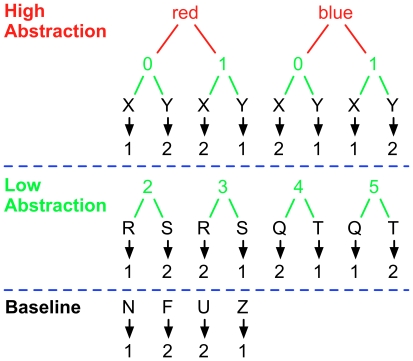
Nesting task structure. In the baseline condition (bottom row), participants were required to select a response to a particular letter stimulus (illustrated in black). One indicates index finger response, and two indicates a middle finger response. In the low abstraction conditions (middle row), participants must use a prior number cue (illustrated in green) in order to appropriately interpret the subsequent probe letter. In the high abstraction conditions, participants must use an additional color cue (illustrated in red) to interpret the number cue that will then allow them to respond to the final probe letter. Dashed blue lines separate the 3 different nesting conditions. The degree of nesting increases as additional relevant cues are added (compare with Figure 2 of Badre & D'Esposito, 2007 [9]). The low abstraction conditions require the control processes in the baseline condition plus those involved with processing the number cue. The high abstraction conditions require the processes in the low abstraction conditions plus those involved with processing the color cue.

**Figure 2:**
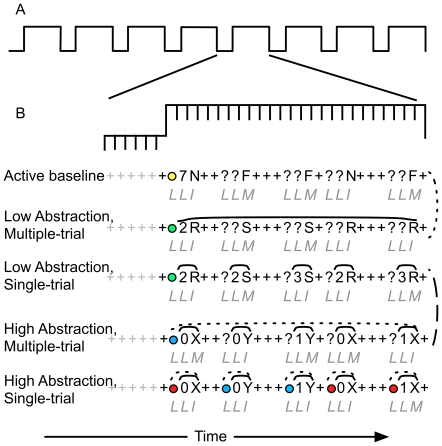
Task Design. Panel A corresponds to the structure of each task within a single BOLD run. Panel B illustrates the 5 conditions. In the low abstraction conditions, participants responded to an imperative stimulus on the basis of a single previously encountered number stimulus (see Figure 1). In the high abstraction conditions, participants responded to an imperative stimulus on the basis of two previously encountered cues (a number and a colored circle). In the multiple-trial conditions, participants received one relevant piece of information at the beginning of a block, and were required to use that information on multiple trials. In the single-trial conditions, participants received every piece of information they needed on a trial-by-trial basis. Italic letters below each stream of stimuli correspond to the appropriate responses: L: left index finger, I: Right index finger, M: Right middle finger. Solid lines over stimuli indicate the maintenance duration of the number cue, whereas the dotted lines over stimuli indicate the maintenance duration of the colored circle. The dashed lines on the right represent conditions in which the trial structure, the number of possible stimuli, and stimulus-response mappings are matched. Taken together, these two contrasts are equivalent to the episodic control contrast (see Figure 3A).

These two hypotheses make similar predictions under many circumstances, since abstract information may systematically need to be maintained for long durations, and concrete information may systematically need to be updated frequently [10]. However, in principle, they can be experimentally dissociated. The first goal of the current study was to investigate which, if either, of these two hypotheses accurately characterizes the recruitment of areas of lateral PFC in a sequential working memory (WM) paradigm. In order to accomplish this goal, we developed an experimental paradigm that orthogonally manipulated two factors. First, we manipulated *maintenance duration*, which was defined as the timing of *when* information-carrying cues were presented and consequently, how long such cue-related information needed to be maintained. Cues were presented either on a trial-by-trial basis or at the beginning of a block of trials (termed single-trial and multiple-trial conditions, respectively). Second, we manipulated *level of abstraction*, which was defined as the degree of nesting, or the number of task-relevant cues that must be processed in order to determine the appropriate action (termed baseline, low abstraction, and high abstraction; see Figures 1 and 2).

**Figure 3:**
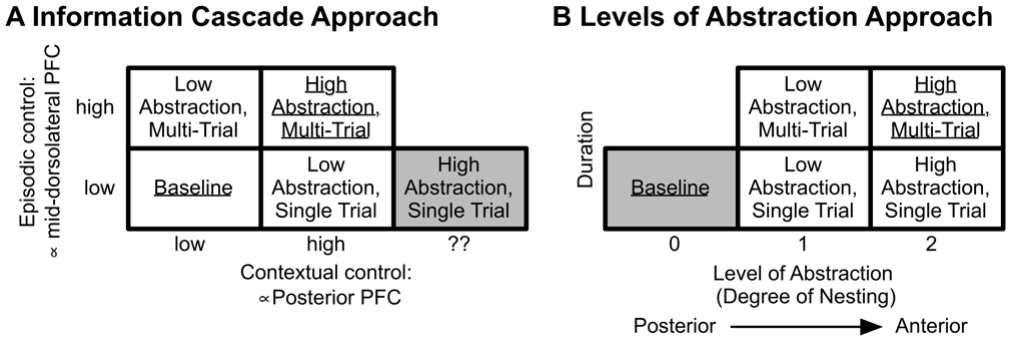
Analysis Approaches. Panel A reflects the analysis that is consistent with the information cascade hypothesis suggested by Koechlin and colleagues [4], [5]. Conditions with information maintained across a block of trials require episodic control and should selectively recruit mid-DLPFC. Conditions with a cue that is updated on each trial require contextual control and should recruit posterior PFC. The white cells correspond to a 2×2 factorial design. The high abstraction, single-trial condition is under constrained in this theoretical framework, and could depend on different levels of contextual control, depending on one's definition of task-set (see Materials and Methods). Panel B reflects the analysis consistent with the levels of abstraction hypothesis suggested by Badre and D'Esposito [9]. As the degree of nesting increases (see Figure 1), increasingly anterior areas of PFC should be recruited. The low abstraction conditions should recruit posterior PFC, whereas the high abstraction conditions should recruit mid-DLPFC. This analysis suggests a different 2×2 factorial ANOVA, as indicated by the white cells. In both panels, the contrast of underlined conditions was used to identify regions of interest, because both hypothesis predict that it should activate both mid-DLPFC and posterior PFC.

When decomposed into these two dimensions of *maintenance duration* and *level of abstraction*, the two hypotheses make different predictions regarding the engagement of lateral PFC. The information cascade hypothesis predicts that anterior areas such as mid-dorsolateral PFC (mid-DLPFC; see Materials and Methods for the empirical demarcation of the different areas of PFC under consideration) should be recruited selectively by task cues that are presented at the beginning of a block of multiple trials, and are relevant over the entire block (i.e. the multiple-trial conditions). This type of temporally distant, block-oriented control has been termed *episodic control* [4], [5]. Conversely, the information cascade hypothesis predicts that more posterior areas of PFC will be recruited by task cues that are presented on a trial-by-trial basis (i.e. single-trial conditions). This type of immediate, trial-oriented control has been termed *contextual control* [4], [5] and, as with episodic control, we adopt this term for consistency with the existing literature. In the current paradigm, these two types of control form a clear 2 (low vs. high contextual control)×2 (low vs. high episodic control) factorial design that can be used to test predictions of the information cascade hypothesis (see Figure 3A): The information cascade hypothesis predicts that more anterior areas such as mid-DLPFC should be sensitive to only episodic control, whereas posterior areas of PFC should be sensitive to contextual control as well as episodic control. The latter effect is predicted from the hypothesized projection from mid-DLPFC to posterior PFC, and thus reflects an anterior-to-posterior cascade of episodic control.

To test the levels of abstraction hypothesis, we used an alternate analysis strategy, and focused on a different set of predictions. The abstraction hypothesis predicts that the critical organizing factor for lateral PFC is the degree of nesting (see Figure 1 and 3B). Specifically, the hierarchical level of task cues should map onto the anterior-posterior gradient of PFC activation, with low-abstraction conditions selectively engaging posterior PFC relative to baseline, and high-abstraction conditions further engaging more anterior regions of PFC relative to the low-abstraction conditions (i.e. mid-DLPFC; see Figure 1 and 3B). Posterior PFC regions are thus predicted to be active in both high and low-abstraction conditions relative to baseline, while mid-DLPFC regions should be engaged only in the high-abstraction conditions. In contrast to the information cascade hypothesis, the levels-of-abstraction hypothesis suggests that the degree of nesting is the relevant factor, irrespective of when contextual cues are presented (see Figure 3B). Thus, the levels-of-abstraction hypothesis predicts the maintenance duration factor should be irrelevant.

The two theoretical accounts make distinct predictions regarding which experimental factors should engage mid-DLPFC and posterior PFC. Thus, the first goal of the current study was to determine whether either hypothesis accurately predicted the activation patterns observed in posterior and anterior PFC regions. In light of this goal, it is important to note that the experimental conditions used in the current study enabled separate tests of the predictions from each theoretical account, because the manipulations used here strongly parallel the manipulations used in prior studies. For example, the baseline and low abstraction, multiple-trial conditions correspond to blocks in prior “motor” experiments used to examine episodic control in tests of the information cascade hypothesis [4], [8]: Each individual trial consists of only an imperative stimulus that requires a decision, but the stimulus-response (S-R) mapping of that decision can be varied across blocks (e.g., in one block, an “R” stimulus maps to an index finger response, and in another, it maps to a middle finger response; see Figure 2). Similarly, the low abstraction, single-trial and high abstraction, multiple-trial conditions correspond to blocks in prior “task” experiments used to examine both contextual and episodic control [4], [8]: Each individual trial includes a cue that provides contextual information regarding the appropriate S-R mapping, but the appropriate S-R mapping can be varied across blocks (e.g. in one block, a “0” stimulus indicates that one should respond to an “X” with an index finger and a “Y” with a middle finger, but in another block, a “0” indicates the reverse mapping; see Figure 2). The additional fifth condition used here (the high abstraction, single-trial condition) does not correspond to a condition used in prior information cascade studies, but does parallel prior “dimension” manipulations used in studies of the levels-of-abstraction hypothesis to create high-abstraction conditions [9]. In such cases, there are three relevant stimuli on each trial, one of which specifies the S-R mapping rule (i.e., dimension) to apply when evaluating the other two.

An additional goal of the experiment was to test a third, qualitatively distinct alternative account of lateral PFC function that relates not to anterior-posterior organization, but rather to the temporal dynamics of PFC engagement:

3. The *adaptive context maintenance hypothesis:* The temporal dynamics of both anterior and posterior PFC adjust dynamically according to strategic and task-dependent maintenance demands. Areas of both mid-DLPFC and posterior PFC are predicted to show sustained activity dynamics when information must be maintained across multiple trials and more transient activity patterns when information must be updated frequently.

This hypothesis synthesizes a large literature demonstrating that the internal representation and active maintenance of contextual information can engage areas of anterior and posterior PFC, even under conditions that involve neither a high level of abstraction nor maintenance demands that span multiple trials [11]–[18]. Here we suggest that the maintenance duration manipulation used in the current study might influence the temporal dynamics of PFC activation, rather than the anatomical location of the PFC regions engaged. Specifically, the adaptive context maintenance hypothesis predicts that lateral PFC activity would be transient in nature (i.e., event-related) under conditions in which a contextual cue is presented and utilized promptly (i.e., single-trial conditions). Conversely, the adaptive context maintenance hypothesis predicts that lateral PFC activity – in the same regions – should be sustained (rather than transient) under conditions in which contextual information was presented at a point in time temporally distal to when it was used, and thus had to be maintained over multiple sequential trials (i.e. multiple-trial conditions). The flexible nature of PFC activation dynamics accords well with the recent Dual Mechanisms of Control framework [19], which postulates that the temporal dynamics of PFC activity flexibly adapts to the relevant control demands. In prior work testing this framework, shifts from transient to sustained PFC dynamics have been observed in relationship to shifts in task control demands or participant control strategy [11], [19]–[21]. Thus, the framework would also predict that a shift in the nature of context maintenance demands (i.e., from single-trial to multiple-trial), would also lead to a shift in PFC activation dynamics.

The focus on PFC temporal dynamics is a strong departure from both the information cascade and levels-of-abstraction hypotheses, which have primarily identified PFC regions either through the use of blocked contrasts or identification of event-related activation. Instead, the adaptive context maintenance account requires analyses that can simultaneously assess and decompose transient and sustained activation dynamics. With such an analysis approach, two testable predictions can be made. First, both posterior and anterior PFC regions should have increased activity in the four experimental conditions that require the internal representation and maintenance of context information (relative to the baseline condition that does not involve context information). Second, this increased activity should have a specific temporal profile. The single-trial conditions that require only transient, within-trial context maintenance should be associated with primarily event-related, rather than sustained, increases in PFC activation. In contrast, the multiple-trial conditions that require maintenance of contextual signals across a block of trials should be associated with sustained, rather than event-related, increases in PFC activation. In other words, PFC activity dynamics should change as a function of maintenance duration. It is important to note that the adaptive context maintenance hypothesis does not postulate that these effects will show a specific anterior-posterior gradient, as previous studies have associated context maintenance with both posterior and anterior regions of PFC. Further, the adaptive context maintenance hypothesis does not differentiate between types of contextual information (such as high vs. low abstraction), and therefore the effects are predicted to be present for both high and low abstraction contexts. In order to investigate the adaptive context maintenance hypothesis, all conditions were run using a mixed block/event-related approach that permits appropriate decomposition of task-related activity into sustained and event-related components (see Methods and Materials) [22].

We next describe the results of analyses testing the predictions made by the three hypotheses concerning the organization and temporal dynamics of lateral PFC. To preview, the observed results regarding the location and pattern of activated lateral PFC regions were not consistent with predictions made by either the information cascade or levels-of-abstraction accounts. However, the temporal dynamics observed in large areas of lateral PFC were in strong accord with predictions made by the adaptive context maintenance hypothesis.

## Results

### Behavior

Overall performance was very high (mean error rates 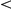
5%, see Table 1). Error rates in the baseline condition (a condition that did not require any contextual or episodic control and had no nesting, see Figure 2) were lower than those observed in all other conditions [*F*(1,28) = 7.5, *p* = 0.01, 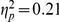; see Table 1]. No other contrast revealed significant differences in error rates [all *F*(1,112)
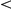
1]. Analyzing the RT data according to the information cascade account revealed a significant main effect of contextual control [*F*(1,112) = 11.9, *p* 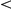
0.001, 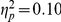], as well as an interaction of contextual control and episodic control [*F*(1,112) = 5.3, *p* = 0.02, 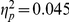]. Analyzed according to the levels-of-abstraction account, significant effects of abstraction [*F*(1,112) = 16.4, *p* 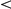
0.001, 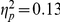] and maintenance duration [*F*(1,112) = 4.2, *p* = 0.04, 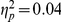] were observed. However, these effects were in a potentially surprising direction, in that they were due to a trend for faster RTs with higher contextual control and abstraction (see Table 1).

**Table 1:** Behavioral error rates and response times.

	% Error	Cue RT	Probe RT
Baseline	1.8 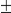 0.5	1002 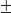 40	580 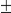 21
Low Abstraction, Multiple-trial	4 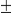 0.9	985 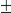 40	611 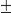 26
Low Abstraction, Single-trial	4.2 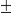 1	1058 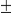 44	567 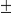 30
High Abstraction, Multiple-trial	3.8 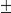 1.1	1027 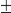 49	543 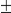 20
High Abstraction, Single-Trial	4.4 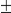 1.2	1029 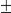 45	539 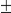 18

Note. Cue RT corresponds to the summed RT across both prior cues for each trial. Uncertainty in each column reflects the s.e. of the mean.

To better understand this pattern, we took advantage of the fact that participants were required to respond to both episodic and contextual cues as well as to probe stimuli, a feature that has been missing from prior investigations of the information cascade and levels-of-abstraction hypotheses. This permitted a test of whether some of the increase in probe RTs observed in prior experiments might have actually reflected the encoding, updating and maintenance of contextual information provided by cues. According to the adaptive context maintenance account, single-trial conditions would involve context updating on each trial, and such processes should be reflected in longer cue RTs compared not only to the (no context demand) baseline, but also to the multiple-trial conditions (in which context cues serve as just a place-holder and convey no information). We tested this hypothesis by taking the summed RT for each of the two cues (or place-holders) present on every trial. Consistent with the adaptive context maintenance hypothesis, participants were slower to respond to cues in the single-trial conditions (mean: 1,043 ms) relative to the baseline conditions [mean = 1,002 ms; *F*(1,28) = 3.97, *p* = 0.056, 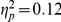], and the multiple-trial conditions [mean = 1,006 ms; *F*(1,28) = 6.51, *p* = 0.02, 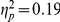]. Such results suggest that previously identified effects are due, at least in part, to encoding, updating, and maintenance processes associated with the presentation of additional information at the time of the response.

### Functional Imaging

The first stage of analysis involved the identification of candidate regions of interest (ROIs) within lateral PFC sensitive to contextual control, episodic control, level of abstraction, or adaptive context maintenance. Because all three theoretical accounts predict that activation in lateral PFC should be increased in the high abstraction, multiple-trial condition relative to baseline, this was used as an unbiased first-stage contrast that enabled us to “cast a wide net” (see Methods and Materials, and Figure 3). Nevertheless, as described further below, a number of control analyses examined alternative contrasts in order to make sure that we had appropriate sensitivity to detect potential PFC regions predicted by the different accounts.

**Figure 4:**
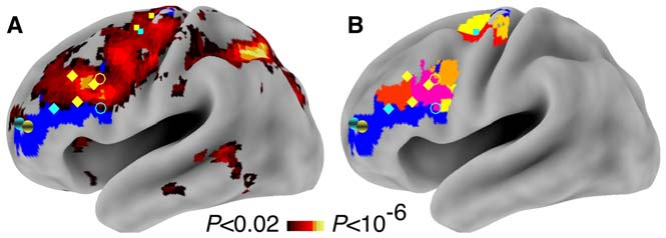
Identified voxels and ROIs. Panel A presents the voxels within the entire brain that showed an increased response in the high abstraction, multiple-trial condition relative to the baseline condition at a voxel-wise 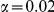
(in order to represent the full extent of activation to this contrast). Panel B represents the ROIs that were created by performing a cluster-level alpha correction within the *a priori* mask, and then separating clusters into component ROIs based on local maxima. In both panels, the blue area represents voxels in the *a priori* mask of lateral PFC that were not responsive. In both panels, the previous peaks from Badre & D'Esposito, and Koechlin and colleagues are represented via symbols. Cyan symbols correspond to peaks reported by Koechlin and colleagues [4], [52], and yellow symbols correspond to peaks reported by Badre &D'Esposito [9]. Small squares represent sensory control or response selection. Open circles represent contextual control or feature selection. Diamonds correspond to episodic control or dimension selection. Spheres correspond to branching or context selection (branching coordinate taken from [52]).

The identification contrast revealed two large clusters of activity in an *a priori* mask of left lateral PFC (see Figure 4): one in dorsal premotor cortex (PMd; center of mass: −29, −8, 58; volume: 9,180 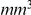), and the other along the upper bank of the inferior frontal sulcus (IFS; center of mass: −43, 16, 30; volume: 10,125 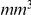). These clusters (particularly the one along the IFS) potentially spanned multiple distinct areas predicted by the information cascade and levels-of-abstraction accounts, with distinct peaks near each of the predicted areas (see Figure 4). Therefore, each large cluster was broken into component ROIs by assigning voxels within the cluster to the nearest peak (see Materials and Methods). This clustering algorithm led to the identification of seven ROIs; three ROIs were identified within the PMd cluster, and four were identified within the IFS cluster (see Table 2). Each of these ROIs were then subjected to follow-up analyses. The first set of analyses utilized block-based comparisons to test anatomical predictions made by the information cascade and levels-of-abstraction hypotheses. The second set of analyses decomposed blocked activity into sustained and event-related responses in order to determine whether such components corresponded to the predictions of the adaptive context maintenance hypothesis.

**Table 2:** Identified frontal regions, ordered from most anterior to most posterior.

						Context	Episode	Low-Base	High-Low	Duration	
Cluster	BA	X	Y	Z	Volume	Blocked	Blocked	Blocked	Blocked	Sustained	Transient
Middle Frontal Gyrus/Inferior Frontal Sulcus (IFS)											
	9/46†	−44	33	28	2214	0.10 ***	0.09**	0.12 ***	0.05	0.03	−0.07
	9/44	−44	17	30	3888	0.08 **	0.10**	0.09 **	0.07**	**0.21** *	**−0.10**
posterior PFC											
	9§	−40	6	37	2187	0.06*	0.07**	0.06*	0.07**	0.12	−0.06
	44‡	−45	5	25	1782	0.06*	0.08**	0.11**	0.03	**0.14**	**−0.12** ***
dorsal Premotor Cortex (PMd)											
	6	−22	−5	60	3132	0.07**	0.08**	0.03	0.07**	**0.16**	**−0.12** **
	6	−34	−7	55	3726	0.08**	0.12***	0.07	0.09***	**0.22** *	**−0.12** **
	6	−33	−14	62	1863	0.12***	0.12**	0.11**	0.10**	0.19	−0.15**

Note.

*P 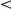
0.10,

**P 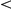
0.05,

***P 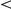
0.01. X, Y, and Z correspond to the coordinates in Tailarach stereotactic space of the center of mass of the ROI, with positive values referring to regions right of (X), anterior to (Y), and superior to (Z) the anterior commissure (AC). Volume refers to the number of voxels (converted to 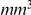) for each ROI. Each of the Context, Episode, Low-Base, and High-Low columns reflect the mean % signal change and significance for the blocked contrasts as defined in Figure 3: Context reflects (high contextual control - low contextual control), Episode reflects (high episodic control - low episodic control), Low-Base reflects (Low abstraction - baseline), and High-Low reflects (high abstraction - low abstraction). Underlined values correspond to significant effects that are not predicted by either the information cascade or levels of abstraction hypotheses. No ROIs demonstrated a significant duration contrast or an interaction among these contrasts. Sustained and Transient columns reflect the mean % signal change and significance for the Duration (multiple-trial - single-trial) contrast on sustained and event-related estimates, respectively (a negative sign indicates greater activity in single-trial conditions). Bold font indicates regions that demonstrated a significant interaction between maintenance duration and temporal dynamics.

† indicates the closest ROI to the mid-DLPFC area identified by an episodic control manipulation in Koechlin, Ody, & Kouneiher, 2003 (distance = 8.1 mm) and any of the mid-DLPFC ROIs identified by the dimension contrast in Badre & D'Esposito, 2007 (distance = 10 mm).

‡ indicates the closest ROI to the posterior PFC area identified by contextual control in Koechlin, Ody, & Kouneiher, 2003 (distance = 5.9 mm).

§ indicates the closest ROI to the posterior PFC ROI identified by the feature contrast in Badre & D'Esposito, 2007 (distance = 5.4 mm).

#### Information Cascade Hypothesis

**Figure 5:**
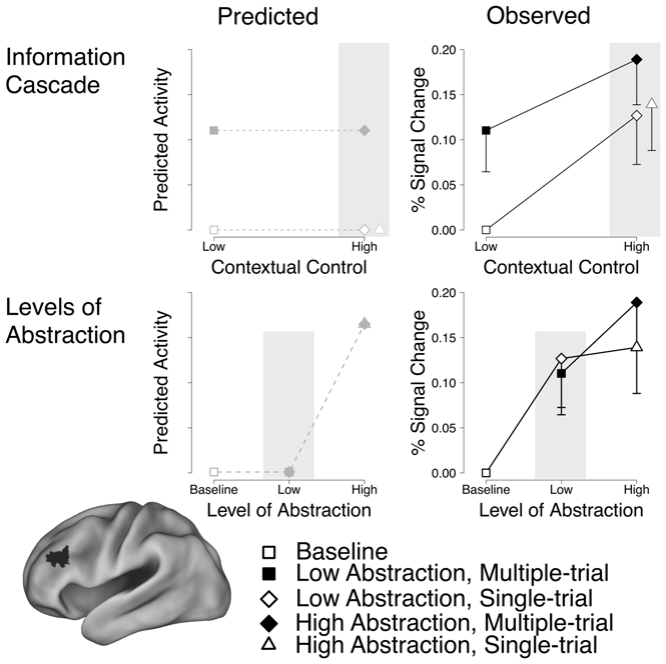
Predicted and Observed blocked activity in mid-dorsolateral PFC. The top row corresponds to predicted (left) and observed (right) activity in mid-DLPFC according to the information cascade hypothesis. Mid-DLPFC (center of mass: −44, 38, 28) was predicted to show sensitivity only to conditions with episodic control demands (left), but also demonstrated sensitivity to contextual control demands (right). The second row corresponds to predicted (left) and observed (right) activity in mid-DLPFC according to the levels-of-abstraction hypothesis. Mid-DLPFC was predicted to show sensitivity only to conditions with the highest level of abstraction (left), but also demonstrated sensitivity to lower levels (right). Gray symbols and dashed lines represent predicted activity (left), whereas black symbols and solid lines represent observed activity (right). Filled symbols represent conditions with high episodic control demands (i.e. the multiple-trial conditions), and open symbols represent conditions with low episodic control demands (baseline and single-trial conditions). Differences between observed and predicted activity are highlighted on a gray field in both rows. Both rows present the same observed data in the right-most column; the data are plotted differently in the two rows to most faithfully represent the hypotheses under investigation and to parallel the organization of Figure 3.

The information cascade hypothesis suggests that the current paradigm should be analyzed with a 2 (low vs. high contextual control)×2 (low vs. high episodic control) ANOVA (see Figure 3A and Materials and Methods). As described above, this hypothesis predicts that all identified ROIs should be sensitive to episodic control, because all of the identified ROIs are inclusive of, or posterior to, the area thought to be responsible for episodic control (mid-DLPFC). The data were consistent with this prediction, as every ROI demonstrated increased activation in the conditions that required episodic control relative to those that did not [min *F*(1,28) = 4.28, *p* = 0.05, 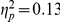; see Table 2]. In contrast, the information cascade approach predicts that the most anterior areas of PFC (mid-DLPFC) should *not* be sensitive to contextual control, while all areas posterior to it should be. The data were inconsistent with this prediction: All ROIs demonstrated at least a marginally significant effect of the context manipulation [min *F*(1,28) = 2.92, *p* = 0.10, 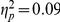], with the weakest effects of contextual control occurring in the two ROIs in posterior PFC (see Table 2). Rather than showing a *null* effect of context, the most anterior ROI identified, and the ROI closest to the coordinates reported for the episodic control by Koechlin et al [4] (current center of mass lay 8.1 *mm* from the mid-DLPFC peak reported previously), showed the *largest* effect of contextual control [*F*(1,28) = 8.6, *p* = 0.007, 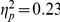, see Figure 5].

One concern over this analysis strategy is that, while the analysis might have been unbiased with respect to identifying voxels sensitive to either contextual control *or* episodic control, it may have had increased sensitivity for detecting regions showing *both* forms of cognitive control relative to regions showing a selective activation pattern. Thus, the analysis may have been biased against identifying voxels demonstrating a null effect of contextual control, and therefore, biased against identifying the pattern predicted for mid-DLPFC. While this criticism does not explain the identification of voxels in mid-DLPFC that are sensitive to contextual control, there could have been an additional set of voxels that did display the appropriate null effect of contextual control, but were not identified in the previous analysis. In light of this concern, we performed an explicit search within lateral PFC dedicated to finding voxels that demonstrated a significant effect of episode, but no effect of context (both *p* 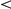
0.02, uncorrected). Two clusters of activity were found to meet the criteria with an appropriate false-positive corrected significance level (cluster 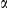 level of 0.05). One cluster fell within posterior PFC (center of mass: −43, 13, 30; volume: 2322 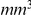), while the other fell within PMd (in contrast to the predictions of the information cascade hypothesis; center of mass: −34,−8,60 volume: 1647 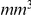). Analysis of these clusters identified two effects of interest. First, of the 147 voxels identified with this analysis, 146 (99.3%) were also identified in the initial analysis, suggesting that the initial analysis had sufficient power to detect voxels that were sensitive to the individual types of control signals, and were not simply identifying voxels that were responsive to both control processes. Second, despite the fact that the voxels were thresholded to demonstrate a null effect of contextual control, cluster-level ANOVAs demonstrated significant effects of contextual control in both clusters [min *F*(1,28) = 4.11, *p* = 0.05], suggesting that the effect of contextual control may be smaller than the effect of episodic control in these clusters, but it is still consistently present.

A second concern is that while all ROIs demonstrated sensitivity to both factors (and no presence of an interaction between the two: max *F*(1,28) = 2.17, *p* = 0.15), it is possible that the current study was simply more powerful than prior studies (the current study used 29 subjects, as compared to 12 in [4] and 19 in [9]), and that the prior studies simply did not have sufficient power to identify the (potentially) relatively smaller effects of contextual control. If this were true, then the identified ROIs still might demonstrate differential sensitivity to each of the manipulations, such that mid-DLPFC might show greater activity associated with episodic control than contextual control, and posterior PFC might show greater responses associated with contextual control than episodic control. We investigated this explicitly by using ROI as a factor in an ANOVA, and testing for ROI x contextual control and ROI x episodic control interactions. ROI did not interact with either of these factors [max *F*(6,168) = 1.09, *p* = 0.37]. Further, when we selected the ROI corresponding most closely to mid-DLPFC (which should be more sensitive to episodic control than contextual control; see Table 2 and Figure 3A) and the ROI corresponding most closely to posterior PFC (which should be more - or equally - sensitive to contextual control than episodic control; see Table 2), there was still no indication of differences across these regions [max *F*(1,28) = 1.95, *p* = 0.17]. While the maximum *F* statistic for the ROI x contextual control interaction came closer to significance (although it did not even reach marginal status), the pattern of the effect was the *opposite* of that predicted: the more anterior ROI trended towards being more sensitive to the contextual control factor than did the more posterior ROI. As such, it is difficult to interpret this null effect as a lack of statistical power (see Analysis S1 for a parallel investigation of decomposed sustained and transient activity).

To summarize, the first set of analyses identified a set of ROIs in lateral PFC that were responsive to both contextual and episodic control. Despite the identification of these ROIs, the data did not support the information cascade hypothesis. Significant effects of contextual control were observed in mid-DLPFC (see Figure 5), in direct contradiction to the predictions of the information cascade hypothesis. In fact, the opposite of the predicted pattern was observed, in that mid-DLPFC showed the strongest (rather than the weakest) effects of contextual control.

#### Levels of Abstraction Hypothesis

In order to investigate whether the levels of abstraction hypothesis provided a better characterization of the data than the information cascade hypothesis, we performed the analysis suggested by the levels of abstraction hypothesis (see Figure 3B). If level of abstraction is the relevant variable underlying lateral PFC organization, then the conditions should be grouped differently than the grouping suggested by the information cascade hypothesis: the condition with 0 degrees of nesting (i.e. baseline) should be isolated, all conditions with 1 degree of nesting (i.e. all low-abstraction conditions) should be grouped together, and all conditions with 2 degrees of nesting (i.e. all high-abstraction conditions) should be grouped together (see Figure 3B). Based on such a grouping, we can define an alternate 2 (abstraction: low vs. high abstraction)×2 (duration: single trial vs. multiple trial) ANOVA from that used in the previous analysis (see Figure 3). Because the levels of abstraction hypothesis also predicts that the high abstraction, multiple-trial condition should elicit greater activity than baseline, the ROIs from the previous investigation were re-analyzed according to this particular approach.

The levels of abstraction hypothesis predicts that posterior PFC should be most sensitive to the lower degrees of nesting (i.e. low abstraction vs. baseline) and that mid-DLPFC should be sensitive *only* to the highest degrees of nesting (i.e. high abstraction vs. low abstraction). In the most posterior cluster identified (within PMd), all three ROIs demonstrated an increased response in the high abstraction conditions relative to the low abstraction conditions [min *F*(1,28) = 6.6, *p* = 0.02, 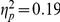, see Table 2]. Within the IFS cluster, two of the four identified ROIs demonstrated an increased response in the high abstraction condition relative to the low abstraction condition [min *F*(1,28) = 4.4, *p* = 0.04, 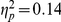; see Table 2]. The two ROIs that did not show an effect of abstraction were identified in the analysis procedure because both low and high abstraction conditions demonstrated increased responses relative to baseline (see Table 2 and Figure 5). No areas demonstrated a difference between single- and multiple-trial durations [max *F*(1,28) = 2.0, *p* = 0.17], or an interaction between the duration and abstraction manipulations [max *F*(1,28) = 1.4, *p* = 0.25].

As stated above, the abstraction hypothesis makes two predictions regarding the functional organization of lateral PFC. First, mid-DLPFC should show increased activation in the high abstraction conditions relative to both the low abstraction and active baseline conditions, but critically, no difference between the low abstraction conditions and baseline (see Figure 5). Second, posterior PFC should show an increased response in both the low and high abstraction conditions relative to baseline. Because posterior PFC and mid-DLPFC are predicted to show different responses in only the low-abstraction condition (posterior PFC should be more responsive in this condition than mid-DLPFC), the contrast between the low abstraction conditions and baseline forms the most diagnostic test for whether there is a gradient within lateral PFC: the levels of abstraction hypothesis predicts that this contrast should be largest in posterior PFC and smallest mid-DLPFC.

All of the (posterior and anterior) ROIs from the IFS cluster showed at least marginally significant increased activation in the low abstraction conditions relative to baseline [min *F*(1,28) = 3.4, *p* = 0.08, 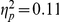, see Table 2 and Figure 5]. Critically, formal tests for differences in this contrast across the identified ROIs revealed no such difference [i.e. no ROI x level of abstraction interaction, max *F*(3,84) = 1.68, *p* = 0.18]. In contrast to the predicted pattern, inspection of individual ROIs revealed that this was not a power issue, as the most anterior ROI demonstrated the *largest* difference between the low abstraction and baseline conditions [*F*(1,28) = 8.0, *p* = 0.01, 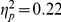, see Figure 5], while one of the posterior PFC ROIs (center of mass: −42, 4, 38) showed the smallest difference between them [*F*(1,28) = 3.4, *p* = 0.08, 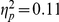]. This is inconsistent with the predicted pattern: more anterior areas should show smaller differences between the low abstraction and baseline conditions. Additionally, the response to the low abstraction conditions identified within mid-DLPFC alleviates one concern with respect to the calibration of the nesting manipulation. The most likely criticism based on such a concern would be that the current high abstraction condition may not be sufficiently abstract to recruit mid-DLPFC relative to the low abstraction condition, and that both conditions would be predicted to activate posterior PFC. Instead, mid-DLPFC appears to be particularly sensitive to the low abstraction condition; if anything, this pattern would suggest that the low abstraction conditions are too abstract to recruit posterior PFC selectively. This interpretation seems unlikely, given that the low-abstraction tasks do not correspond to prior definitions of abstraction.

As with the analysis based on the information cascade approach, it is important to rule out whether the lack of support for the abstraction hypothesis is due to any potential biases or confounds in the analysis approach. One potential bias is that the identification procedure could have low sensitivity for detecting voxels that exhibited increased activity in the high abstraction condition relative to the low abstraction condition, but no difference between the low abstraction condition and baseline. In order to ensure that the effects seen were not due to low sensitivity effects, we performed a search dedicated to finding voxels that demonstrated a significant difference between high and low abstraction, but no difference between low abstraction and baseline. One cluster of activity was found to meet these criteria with an appropriate false-positive corrected significance level (cluster 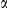 level of 0.05). This cluster fell in PMd (center of mass: −29, −4, 57; volume: 4779 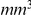). Similar to the PMd ROIs identified above, it demonstrated no difference between the low abstraction conditions and baseline [*F*(1,28) = 1.7, *p* = 0.20], and a difference between the high and low abstraction conditions [*F*(1,28) = 12.7, *p* 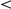
0.001]. As we found with the information cascade analysis, almost all of the voxels in this region were also identified in the original analysis (163/177 voxels: 92%), suggesting that the original analysis was sensitive enough to detect such patterns. Critically, the location of the ROI was too posterior and superior to be consistent with the abstraction hypothesis, and no clusters in more anterior regions of PFC (i.e. mid-DLPFC) were identified.

Another potential concern that is important to rule out is whether the statistical tests masked a true pattern of differential sensitivity to the two levels of abstraction within anterior and posterior areas of lateral PFC. For example, even though mid-DLPFC might demonstrate sensitivity to the low-abstraction condition relative to baseline, it may be the case that this effect is smaller than the same effect in posterior PFC. To test the validity of this concern, we investigated whether the strength of the low abstraction vs. baseline contrast varied systematically with ROI location. Across all seven ROIs, there was no difference in the strength of the contrast [*F*(6,168) = 1.2, *p* = 0.33]. If we compare only the two ROIs that are closest to those previously reported (see Table 2), then the *F*-statistic for the interaction becomes larger [*F*(1,28) = 2.5, *p* = 0.12], but as in the previous information cascade analysis, the direction of the interaction is in the opposite direction than that predicted by the abstraction hypothesis, with the posterior PFC ROI demonstrating less sensitivity to the low abstraction condition than the more anterior mid-DLPFC ROI (see Analysis S1 for an investigation of decomposed sustained and transient activity).

To summarize both sets of analyses, neither of the two hypotheses regarding the functional organization of lateral PFC (information cascade and levels of abstraction) were able to adequately characterize the pattern of activity observed in the current experiment. Therefore, we sought to investigate whether the adaptive context maintenance hypothesis could provide a better account.

#### Adaptive Context Maintenance Hypothesis

The adaptive context maintenance hypothesis makes two specific predictions, the first of which has already been investigated above. Its first prediction is that areas of lateral PFC (including anterior areas such as mid-DLPFC) should be active in all conditions relative to baseline, because these conditions have an additional context maintenance demand. The prior two analysis procedures have already addressed this prediction: areas in mid-DLPFC, posterior PFC, and PMd all demonstrated increased activity in conditions requiring maintenance relative to baseline, irrespective of whether the to-be-maintained information reflected contextual control, episodic control, low abstraction levels, or high abstraction levels (see Table 2). The identification of areas in mid-DLPFC is particularly notable, as they were predicted by the adaptive context maintenance hypothesis, but not by either of the two other hypotheses (see Table 2 and Figure 5). Second, and more importantly, the adaptive context maintenance hypothesis predicts that this activity should have a specific temporal profile that varies with task demands: activity is predicted to be transient in conditions in which contextual information is updated on each trial, but sustained in conditions requiring maintenance of contextual information across a block of trials. Further, the adaptive context maintenance hypothesis predicts that these temporal dynamics should be present irrespective of the type of contextual information involved (i.e. low vs. high abstraction). In order to test this more specific prediction regarding activity dynamics, the responses of each ROI were first decomposed into sustained and transient components [22]. Next, the sustained and transient estimates were entered into a single 2 (single vs. multiple-trial maintenance duration)×2 (low vs. high abstraction)×2 (sustained vs. transient activation dynamics) repeated measures ANOVA. Sustained estimates were predicted to demonstrate increased activity in the multiple-trial conditions relative to the single-trial conditions, whereas transient estimates were predicted to demonstrate increased activity in the single-trial conditions relative to the multiple-trial conditions. Such a pattern is reflected in the statistical interaction between the activation-dynamics and maintenance-duration factors. Abstraction was included as a factor in order to assess whether the predicted pattern of temporal dynamics changed as a function of abstraction (as reflected in the 3-way interaction between activation dynamics, maintenance duration, and level of abstraction).

**Figure 6:**
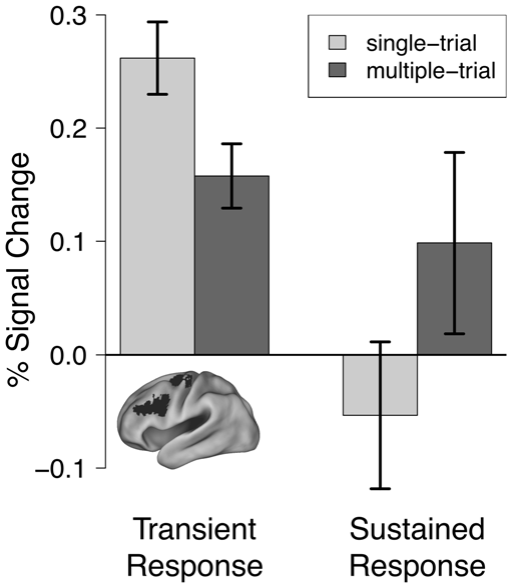
Percent signal change as a function of activity dynamic and maintenance duration manipulation. Y-axis is the % signal change averaged across all identified ROIs. Single-trial conditions were associated with increased transient activation relative to the multiple-trial conditions [left group of bars: *t*(28) = 2.2, *p* = 0.04]. Conversely, multiple-trial conditions were associated with numerically increased sustained activation relative to single-trial conditions [right group of bars: *t*(28) = 1.6, *p* = 0.12]. This cross-over pattern resulted in an activity dynamic x maintenance duration interaction: [*F*(1,28) = 4.0, *p* = 0.05].

Two ROIs within lateral PFC demonstrated a significant maintenance duration X activity dynamics interaction, as predicted by the adaptive context maintenance hypothesis [min *F*(1,28) = 4.72, *p* = 0.04, 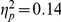; see Table 2], while another two ROIs demonstrated a marginally significant interaction [min *F*(1,28) = 3.34, *p* = 0.08, 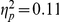]. This effect was consistent across both levels of abstraction across all ROIs (the abstraction x duration x dynamics interaction was not significant: all *F* 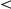
1). Interestingly, the pattern of results across ROIs suggested that all ROIs displayed this pattern at least numerically (see Table 2). This suggestion was investigated via a supplementary ANOVA that included ROI as an additional factor. This analysis revealed a maintenance duration x activity dynamics interaction [*F*(1,28) = 4.04, *p* = 0.05, 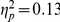; see Figure 6] that did not interact with level of abstraction or ROI [both *F* 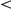
1], indicating that this pattern of activity dynamics was consistent across the identified ROIs. Recall that all of the PFC ROIs were identified from an original contrast that was independent of the analysis of activation dynamics. The results suggest that in the identified regions, the increases in sustained activation found in the multiple-trial conditions were associated with corresponding decreases in transient activation in these conditions. This shift in activation dynamics paralleled the demands on context maintenance. To summarize, the observed pattern of activation dynamics provided evidence that supported the adaptive context maintenance hypothesis.

## Discussion

The current study employed a factorial experimental design that permitted the testing of three hypotheses regarding the function and organization of lateral PFC. In one set of analyses, we tested the information cascade hypothesis [4], [5], which is based on distinctions in the timing of task-related cues: episodic vs. contextual (i.e. multiple-trial control vs. single-trial control). In another set of analyses, we tested the levels of abstraction hypothesis [1], [9], which is based on the degree of hierarchical nesting of control signals (it should be noted that and other operational definitions of abstraction such as those used in [23], [24] might produce different results). These analyses identified a set of lateral PFC ROIs broadly sensitive to episodic control and high abstraction. More importantly, however, they indicated that the profile of activity in these regions was incompatible with that predicted by either hypothesis. Specifically, anterior regions of mid-DLPFC exhibited greater activity to both contextual control and low abstraction conditions than did areas of posterior PFC, even though these patterns are opposite to the patterns predicted by the two hypotheses. We proposed a third hypothesis as an alternative, the adaptive context maintenance hypothesis, and identified several findings in support of it. In particular, we observed that activity appeared to shift between transient and sustained patterns according to the duration over which task information had to be actively maintained. While this pattern was predicted by the adaptive context maintenance hypothesis, it was not predicted by the other two hypotheses. In the following sections, we discuss implications of the current findings for the wider literature on lateral PFC organization and function.

### Maintenance demands as an explanation of prior results

One key result of the current experiment is that mid-DLPFC can be recruited under circumstances that require context maintenance, even if such demands are quite short, and even if the information that needs to be maintained is not particularly abstract. One potential explanation for this observation, and those of prior studies, is that areas of mid-DLPFC (and other areas of lateral PFC) are sensitive to conditions that include some demand for context maintenance as an opportunity for preparatory processing. Further, this explanation is also consistent with a large computational modeling literature suggesting that DLPFC, in particular, is important for the maintenance of context information that can bias on-going activity in other areas of cortex [7], [25]–[27]. A recent computational model attempted to relate the hierarchical organization of PFC to distinct mechanisms associated with active maintenance and updating of information in WM using a task similar to that described here [7]. The model demonstrated a graded degree of hierarchical organization, with some simulated neurons exhibiting a hierarchical pattern, and others not. Given that that the fMRI BOLD signal aggregates over many neurons in a given area, the different neuron types may not be evident. Further, the mechanisms that govern the updating and maintenance of information in such models predict the pattern associated with the adaptive context maintenance hypothesis. Specifically, the updating of information in WM is dependent upon a phasic signal (thought to be mediated by the basal ganglia and/or dopaminergic input) that dynamically modulates how long information is maintained [27]. Information is effectively maintained until another similar phasic signal is encountered. The presence of such an adaptive gating mechanism runs counter to any organization according to fixed (or even relative) durations of maintenance.

When viewed from this perspective, prior results in the literature on the hierarchical organization of lateral PFC might be explained in terms of context maintenance and preparatory processes. In particular, the information cascade hypothesis has suggested that multiple-trial maintenance is associated with mid-DLPFC, but has not previously tested whether maintenance within a trial is sufficient to activate mid-DLPFC (in the prior studies, context information was presented simultaneously with the probe stimulus, and therefore available at the time of the decision process). Further, the abstraction hypothesis could also potentially confound level of abstraction with context maintenance demands. For example, in prior tests of the abstraction hypothesis, the highest levels of abstraction have also explicitly required the active maintenance of contextual information across multiple trials [9]. For the second-highest level of abstraction, prior investigations have used task conditions that require the processing of three simultaneously presented pieces of information, one of which represents a rule or dimension that must be encoded and then used to select the relevant features in the other two stimuli. It is possible that these task demands are met by encoding and then actively maintaining the rule/dimension, in order to bias the encoding and processing of the additional stimuli [28]. The conditions that probe lower levels of abstraction may involve the use of simple enough rules that response mappings can be directly activated without the need for active maintenance. One way to test this hypothesis would be to manipulate rule complexity and maintenance demands at lower levels of abstraction, to determine whether such manipulations are associated with activation in anterior areas such as mid-DLPFC.

It is important to note that the hypothesis that maintenance demands are important for the recruitment of mid-DLPFC is not inconsistent with the information cascade hypothesis, with the caveat that the maintenance delay does not need to involve long periods of time, nor be extended over multiple trials. One could argue that because all of the cues in the current study occur prior to the probe stimulus, they all serve as episodic control signals, and thus should recruit PFC regions associated with this level of control (i.e. mid-DLPFC). While this is one potential interpretation of the current results, it is at odds with the primary qualitative distinction between episodic and contextual control as formulated in the information cascade hypothesis: episodic control signals should be relevant for multiple trials, whereas contextual signals should be relevant only for a single trial. If an episodic signal is any one that occurs prior to the probe stimulus, then it suggests a very different functional model, in which it is only the SOA between cues and probes that determines the level of control (i.e., contextual control: SOA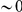; episodic control: SOA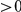). This would seem to be a rather narrow definition of contextual control, relative to what has been implied in prior treatments of the information cascade hypothesis [5], and would likely require revision and reformulation of standard ideas regarding such control processes.

It is worthwhile to further consider potential reasons for the discrepancy between activation patterns observed in the current study and those observed in previous ones examining the information cascade and levels of abstraction hypotheses. As described above, prior studies used manipulations in which contextual and probe information were presented simultaneously rather than sequentially. Further, in such studies, the contextual and imperative stimuli were often presented as an integrated stimulus (e.g. a colored letter, in which the color indicates the task, and the letter provides the imperative information) as opposed to two distinct ones (e.g. a red circle next to a letter). Thus, it is possible that the temporal and spatial segregation in the current study may have placed distinct demands on anterior versus posterior PFC relative to the prior work. Future work will be needed to directly manipulate these two factors to determine if either (i.e., sequential/simultaneous or segregated/integrated presentation) modulates the pattern of activation observed in mid-dorsolateral vs. posterior regions of PFC.

### The role of preparatory processing

The results are largely consistent with the adaptive context maintenance hypothesis, which is based on prior studies investigating active maintenance processes in mid-DLPFC during simple sequential WM tasks [11]–[18]. ROIs supporting this hypothesis were found across the PFC, including areas near the inferior frontal junction (IFJ; BA 6/9/44), mid-DLPFC (BA 9/44), and dorsal pre-motor cortex (PMd; BA 6). Previous work has established that the identified regions (particularly IFJ and mid-DLPFC) are involved in processing contextual cues in a variety of tasks, including Stroop and task-switching paradigms [13]–[17]. The current findings augment this prior work by suggesting the same contextual cues may activate these regions with different dynamics, depending on the temporal demands of context maintenance in such tasks.

A key implication of the current findings is that shifts in the activity dynamics within a variety of lateral PFC regions may reflect a shift between transient and sustained maintenance of contextual information. These shifts appear to represent a change in the engagement of relevant preparatory processes to meet the demands on context maintenance. Such shifts are consistent with recent evidence suggesting that a given area of PFC may be associated with different temporal dynamics depending on a variety of factors, such as motivation and reward [29], cognitive ability [30], and experience [20]. Indeed, the current data fit well with the Dual Mechanisms of Control framework [19], which postulates that lateral PFC activity dynamics can flexibly adapt in accordance to current cognitive control demands. The current findings extend the range of experimental factors that are associated with flexible adaptation of lateral PFC activity dynamics by demonstrating that direct manipulations of the required duration of context maintenance can also cause a shift in activation dynamics within PFC. This is a novel finding, as it demonstrates that temporally extended (i.e. sustained) active maintenance can be observed when the maintenance interval extends across the presentation of multiple intervening task trials and responses (and not just the simple duration of maintenance within a trial, e.g. [31]). By definition, detection of such shifts between transient and sustained patterns requires an analytic approach that can detect and isolate activation that persists across inter-trial intervals from purely transient within-trial activity; such approaches have only recently been gaining popularity in working memory (WM) studies [32]–[34]. Yet, the results are reminiscent of prior neurophysiological work showing persistent maintenance of sample cue information across behavioral responses to intervening distractor stimuli in PFC neurons [35].

Similar to the current results, a large number of prior studies have identified mid-DLPFC activity associated with context maintenance, even when using very simple task cues and structure [13]–[18]. For example, the AX-continuous performance task (AX-CPT) has been used repeatedly to probe mid-DLPFC activation, despite the fact that the task is extremely simple: make a target response if you see an X that follows an A, and make a non-target response otherwise (in fact, the current paradigm is an extension of such a task, and the high abstraction, multiple-trial condition is an implementation of an extension termed the 1/2-AX-CPT [7], [27]). While the information cascade hypothesis would argue that the AX-CPT involves only contextual control, and the abstraction hypothesis would suggest only one degree of nesting (the response to the probe depends on the preceding cue), several studies have identified mid-DLPFC activity in this task [11]–[13]. Moreover, within the AX-CPT, the activation pattern in mid-DLPFC is sensitive to different cue types [11], [13], [36], [37] and predicts behavioral performance [13]. These cue-specific effects suggest that this region is engaged because of different control demands associated with distinct types of contextual cues, such as different maintenance demands, different encoding or detection processes, or differential predictive ability. The AX-CPT paradigm can be viewed as a special case of more generic task-cueing (or task-switching [38]) paradigms in which a prior cue specifies the rule one uses to select an appropriate response. There are also a number of other studies that suggest the recruitment of mid-DLPFC in these types of paradigms [39]–[41]. Therefore, it is unclear how the prior approaches to the anterior-posterior organization would conceptualize these types of preparatory processes. One possibility is that the DLPFC regions identified in prior studies of the AX-CPT and other preparatory processing tasks are distinct from the ones associated with episodic control or higher degrees of nesting. However, the current data would suggest otherwise; the *a priori* mask we used to identify mid-DLPFC was based directly on coordinates taken from the studies identifying episodic control and high-abstraction DLPFC regions. Yet, the ROI that we identified still demonstrated sensitivity to advance contextual cues that had a low degree of abstraction. Thus, a primary conclusion to be drawn from the current work is that the role and functional effects of preparatory processing should be a primary consideration in theoretical accounts of how cognitive control is deployed. In their current form, the information cascade and levels-of-abstraction hypotheses make predictions based completely on task structure, and do not allow for such distinctions. However, as we have argued, theoretical accounts such as the Dual Mechanisms of Control framework, which focuses directly on the role and potential for strategic preparation in the modulation of control processes, may represent a promising direction in this area [19], [20].

### Conclusions

The current experiment provides two critical and novel insights into the function and organization of lateral PFC. First, areas of lateral PFC were recruited in a manner that directly conflicts with predictions made by current conceptions of two prevalent theories (the information cascade and levels-of-abstraction hypotheses). Specifically, mid-DLPFC was strongly associated with low-level increases in abstraction and contextual, as well as episodic, control signals. Second, lateral PFC activation dynamics were modulated by task-demands, such that they were transient when contextual information was presented on a trial-by-trial basis, but sustained when contextual information had to be maintained across multiple trials. In contrast, these results are consistent with the adaptive context maintenance hypothesis, which suggests that the recruitment and dynamics of lateral PFC depend on the demands for context processing along several dimensions, including the need for preparatory processing and the temporal duration over which it extends.

## Materials and Methods

### Participants

Twenty-nine right-handed participants with no evidence of neurological compromise participated in this study. One additional participant was recruited, but did not complete the study due to scanner malfunction. Participants were nine males and 20 females with a mean age of 23 years (age range: 19–34 years). The study protocol was approved according to guidelines set by the Washington University Medical Center Human Studies Committee. All participants provided written informed consent prior to participation, and were paid $25/hour as compensation.

### Behavioral Tasks

Participants performed five conditions of a delayed-response WM task that each involved a continuous series of stimuli, presented one at a time (see Figure 2). Along with a baseline (control) condition, two factors were orthogonally manipulated: 1) the number of trials (maintenance duration) over which information needed to be maintained (or, conversely, how often information needed to be updated; single- vs. multiple-trial); and 2) how abstract the relevant stimulus-response mappings were (low abstraction vs. high abstraction). Similar to previous definitions of abstraction, the operational definition of abstraction was the degree of nesting of the currently appropriate stimulus-response (S-R) mapping rule (see Figure 1) [9]. In the current paradigm, nesting refers to the number of stimuli that need to be considered to determine the appropriate response. In the baseline conditions, participants responded simply on the basis of each letter they saw (e.g., they responded with their index finger to all F's and with their middle finger to all N's, see Figures 1 and 2). Because they only had to consider the letter stimulus to determine their response, this condition was considered to have 0 degrees of nesting In the two low-abstraction conditions, determining the correct response to the probe depended on the value of a previous number cue (a single digit). For example, if the most recent cue was a 2, participants responded with their index finger to an R and with their middle finger to an S, but if the cue was a 3, the response mappings were reversed (see Figures 1 and 2B). Because the appropriate response to each letter was nested within only one other determining factor (the number cue), this condition was considered to have 1 degree of nesting. In the two high-abstraction conditions, the correct response to a probe was based on the value of a previous number cue and an additional previous color cue. For example, if the most recent colored circle was red, then participants responded with their *index* finger to an X that followed a zero or a Y that followed a one. However, if the most recent colored circle was blue, then participants were asked to reverse the mappings, such that they responded with their *middle* finger to an X that followed a zero or a Y that followed a one (see Figures 1 and 2B). Because the appropriate response to each letter was nested within two determining factors (the number cue, which was nested within the colored circle), this condition was considered to have 2 degrees of nesting.

The maintenance duration manipulation referred to how often contextual cues were presented. In the single-trial conditions, all contextual cues were presented on every trial, such that all of the information needed to determine a response to the current probe was randomly updated and presented on a trial-by-trial basis (e.g. in the low-abstraction conditions, participants received a number cue and a probe on every trial; in the high-abstraction conditions, participants received an color cue, a number cue, and a probe on every trial). In the multiple-trial conditions, one piece of information associated with each mapping was updated only at the beginning of a 5 trial block (e.g. the number cue was only presented on the first trial of a block in the low-abstraction, multiple-trial condition, and the color cue was only presented on the first trial of a block in the high-abstraction, multiple-trial condition). To control for stimulus presentation and sequence effects, in the multiple-trial conditions, these cues were replaced with question marks (see Figure 2).

All stimuli were counterbalanced across participants and conditions. Each trial within a block consisted of the presentation of 3 sequential stimuli: 1) a question mark or color cue indicating the start of a trial (both of which required a left hand index finger response – question marks were used in control trials to maintain the same visual stimulation, number of manual responses, and trial timing as in other trials), 2) a question mark or number cue (both of which also required a left hand index finger response), and 3) an imperative probe stimulus (that required a right hand response, either index or middle finger, depending on the probe and the relevant S-R mapping rule). All stimuli were presented on a black background in the center of the screen in 48-point bold Helvetica font. All letters and numbers were presented in white, and all probes were underlined, to signify that they required a different response from the cue items. All stimuli were presented for 300 ms, and the stimulus-onset asynchrony (SOA) between cues and subsequent probes was 2500 ms. Participants were required to respond within 1500 ms of each cue or probe onset. After each probe, a white fixation cross appeared for a variable inter-trial interval (ITI) of 2200 to 7200 ms in order to allow for the estimation of the transient hemodynamic response on each trial [42]. The number of 2500 ms fixation events in the variable ITI had an approximately geometric distribution with 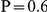.

### Functional Neuroimaging

MR images were acquired on a Siemens 1.5 Tesla Vision System (Erlangen, Germany) with a standard circularly-polarized head coil. A plastic face mask was used to minimize head movement. Headphones were used to dampen scanner noise and enable communication with participants. Both structural and functional images were acquired at each scan. High-resolution (1.25×1×1) structural images were acquired using a sagittal MP RAGE T1-weighted sequence [43]. Functional images were acquired using an asymmetric spin-echo echo-planar sequence (TR = 2500, TE = 50 ms, flip = 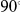). Each image consisted of 18 contiguous, 7 mm thick axial slices acquired parallel to the anterior-posterior commissure plane (3.75×3.75 mm in-plane), allowing complete brain coverage [44].

Each functional scanning run consisted of 8 alternating cycles of task and fixation blocks with an additional fixation block at the beginning of the BOLD run (see Figure 2). The inclusion of fixation blocks enabled the decomposition of sustained and transient hemodynamic responses [22]. The first three images in each scanning run were used to allow the scanner to reach steady state and were therefore discarded. Each BOLD run lasted approximately 9 minutes, and a 3-minute delay between runs gave the participants time to rest. Each run corresponded to one of the 5 conditions described above. Two additional experimental conditions were also scanned, but are not relevant for the current analyses.

### Image Preprocessing

Functional imaging data were preprocessed and analyzed using in-house software. Preprocessing steps included correction for movement using a rigid-body rotation and translation correction [45], [46], registration to the subject's anatomical images (in order to correct for movement between the anatomical and functional scans), temporal realignment using cubic-spline interpolation, intensity normalization (to an arbitrary value of 1000 for each scanning run), resampling into 3 mm isotropic voxels, and spatially smoothing with a 9 mm FWHM Gaussian kernel. Each participants' anatomical volume was transformed into a standardized atlas space [47]–[49] using a 12-dimensional affine transformation [50], [51], and the functional images were then registered to the reference brain using the alignment parameters derived for their anatomical data.

### Behavioral Data Analysis

Mean error rates and median response times (RTs) were investigated to determine whether they differed across conditions. Similar to the imaging data, we performed analyses based on both the information cascade and levels-of-abstraction hypotheses (see Figure 3).

### fMRI Data Analysis

#### Blocked Analyses

A general-linear model (GLM) approach [42] was used to estimate parameter values reflecting the mean difference between the task and fixation blocks for each experimental condition. The blocked analysis procedure was designed to identify areas of lateral PFC for which BOLD activation increased as a function of the maintenance duration and abstraction factors (see below for a description of how these 2 factors relate to the information theoretic model characterizing contextual and episodic control). Because we were primarily interested in understanding how these variables influenced activation levels in previously defined regions of interest (ROIs), we created an initial *a priori* mask by drawing 12 mm spheres around the coordinates reported by two previous studies investigating hierarchical processing in PFC [4], [9] (peaks for fronto-polar cortex were also taken from Koechlin et al, 1999 [52] in order to represent the full information cascade model). Within this initial mask, we identified voxels that demonstrated a significant increase in the theoretically most taxing condition (high abstraction, multiple-trial) relative to the active baseline. Because both the information cascade and abstraction accounts predict increases in the high abstraction, multiple-trial condition, the application of this constraint increases the interpretability of any identified effects without biasing the results to favor either of the tested hypotheses. Within this mask, we identified clusters of activity that were sensitive at a cluster-level 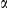 rate of 0.05, as determined by the AFNI tool 3dClustSim (voxel-wise constraints: voxel-wise 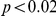, cluster size 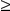
45 voxels; other voxel-wise thresholds and cluster-sizes produced consistent results).

Because the identified clusters were quite large, they were broken into component ROIs by identifying local maxima within the cluster, removing any local maxima within 12 mm of a larger local maximum, and assigning each voxel within the cluster to its nearest resulting maximum. Within the resulting ROIs, we performed two different analyses in order to test the hypotheses regarding the posterior-anterior organization of lateral PFC. The first analysis tested the information cascade account proposed by Koechlin and colleagues [4], [5]. The second analysis tested the levels of abstraction account proposed by Badre & D'Esposito [9]. All of the group level analyses involved computing the average activation level of each ROI for each participant, and submitting each of those to a group-level ANOVA or *t*-test in which participant was treated as a random effect.

#### Information Cascade Approach

We first review the information theoretic framework that formed the basis of the analysis examining different levels of cognitive control within lateral PFC. A stimulus provides *information* (*I*) regarding a variable to the extent that it reduces that variable's uncertainty [53]. The uncertainty (i.e. entropy) of a set of responses (*R*) can be calculated as:
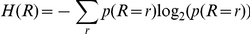
where *r* indexes each particular response, and *p(r)* is the probability of that response being made. In the current paradigm, the amount of uncertainty associated with the probe response in all conditions is 1, because there are always two potential responses, and each response is equally likely [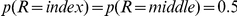].

According to information theory, we can reduce this uncertainty by accumulating information associated with various different stimuli. For example, if one is interested in the amount of information a particular stimulus (*S*) provides about a response, then one can begin by calculating the uncertainty of the response if that stimulus is known 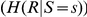. If one is interested in calculating the entropy of the response set across all stimuli, then you aggregate across the relevant stimuli (*s*):
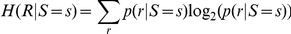


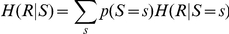
Once that reduced uncertainty is known, the information provided by the stimulus (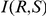) is calculated by subtracting that residual uncertainty from the original measure of uncertainty:
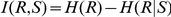
The 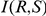 nomenclature is used to be consistent with the information cascade model proposed by Koechlin et al, 2003 [4]. The value actually used in their analyses (termed 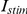), and also used for the analyses in the current study, is the information provided by the stimulus about the response, given that the episode (*U*) is known (i.e. 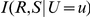). Although not explicitly calculated in their prior explorations of contextual control, one could argue that 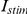 should also be contingent upon any contextual cues (*C*) as well (i.e. 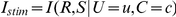). In the current paradigm, doing so indicates that 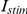 is equivalent across all tasks: each task has the same amount of uncertainty in the response set because two responses are always eligible and they always occur with the same frequency (i.e. 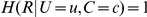 for each combination of *u* and *c*). Further, once the stimulus 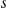 is known, all uncertainty in the response is removed (i.e. 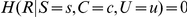). While 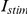 is held constant across conditions in the current study, other stimuli do provide different amounts of information, and these other sources of information form the basis of the investigation of contextual and episodic control. Specifically, the values that vary are the amounts of information provided by the contextual (*C*) and the episode cues (*U*).

The context and episode cues are additional stimuli that determine the task-set (*T*) to be performed. While task sets are potentially complex constructs that have multiple definitions and features [54], we define a task set explicitly in this experiment as the set of probe-response mappings that can be selected once the context and episode cues are known such that 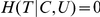. For example, the task-set for a baseline condition (see Figure 2) could be 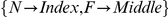. A contextual cue is a stimulus that occurs *on each trial* and provides some information regarding the task-set (i.e. reduces its uncertainty), given that the value of the probe stimulus is encoded:
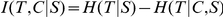
As above, the nomenclature of 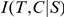 is used to be consistent with Koechlin et al, 2003. The value actually used in their analyses (termed 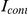), and also used here, is the information provided by the contextual cue about the task, given that the probe stimulus (*S*) and episode (*U*) cues are known: 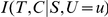. Interestingly, this particular computation could be problematic for paradigms in which a contextual cue is presented prior to a stimulus: the contextual cue *C* is presented prior to the stimulus *S*, and yet the calculation of 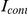 requires that *S* be known. This illustrates a potential problem with generalizability of this particular information cascade approach. However, it does not pose a problem for the current paradigm, as probe stimuli are equiprobable and balanced within an episode, and therefore do not alter the information provided by contextual cue 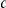 about the currently relevant task set (i.e. 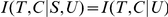). For the baseline and low abstraction, multiple-trial conditions, this value is 0. In both of these cases, the cues that are presented *on each trial* are question marks, and do not help to determine the appropriate response (see Figures 2 and 3). In the cases of the low abstraction, single-trial and high abstraction, multiple-trial conditions, this value is 1. In each possible scenario, there are two potential sets of stimulus-response mappings, they are equally likely (e.g. 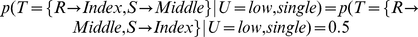), and that uncertainty is completely removed once the contextual cue is known. In contrast, this value for the high abstraction, single trial condition is critically dependent on the definition of task-set, and we return to it after discussing the information provided by the episode (*U*).

The information cascade approach also defines an episode cue. This episodic cue is a stimulus that occurs *at the beginning* of a block of trials, and carries information that is relevant for multiple trials. Specifically, it provides additional information regarding the task-set *T*, given other signals (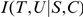. In this definition, it is useful to note an inconsistency in the flow of information: this particular value requires some uncertainty in either the task or episode, while presuming knowledge of the stimulus and context values, while calculation of 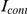 and 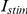 requires uncertainty in *S* and *C*, while presuming knowledge of *U*. Nevertheless, this information value is 0 for the baseline and single-trial conditions, because all information is carried by the stimulus *S* and context *C*, and therefore, there is no uncertainty for the episode 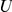 to eliminate. In contrast, this value is 1 for the multiple-trial conditions, because there is residual uncertainty in the current block if the episodic cue appearing at the beginning of the block is not encoded: For example, in the low-abstraction, multiple-trial condition, if the episodic cue was not encoded, it would be impossible to determine which of the two probe-response mappings was relevant (see Figure 2).

These information values create a clear 2 (amount of context information)×2 (amount of episode information) repeated-measures ANOVA (see Figure 3A). Interestingly, the high abstract, single-trial condition falls out of the 2×2 information cascade analysis. The information value associated with contextual control for this condition depends critically on the definition of the task-set (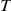). For this task, we assume that these two cues presented on each trial are both considered contextual cues (since contextual cues, by definition, are presented on every trial), and that together, the two individual context cues form a single conjunctive cue. If the task-set is defined as the set of probe-response mappings (as we defined above), then the 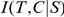 value should be equal to 1, which is equivalent to the low abstraction, single-trial condition. This approach and interpretation is equivalent to suggesting that there is a single 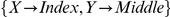 set of probe-response mappings (see Figure 1), and that both the “red-0” conjunctive cue and the “blue-1” conjunctive cues would identify that single mapping as being relevant. As such, there are two equiprobable task sets, and knowing the conjunctive cue completely eliminates uncertainty. Alternatively, if this condition is construed to activate a distinct task-set for each conjunctive cue (irrespective of the fact that the two conjunctive cues activate the same probe-response mapping), then a task set can be redefined explicitly as the possible conjunctions of 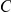 and 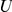 (this redefinition has no bearing on the other 4 conditions; they are all identical in terms of the calculated information values). With this definition, the amount of information provided about the task by the conjunctive contextual cue equals 2, because there are four equally probable alternatives (one for each of the four possible conjunctions of 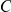 and 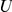), and once the two cues are presented, there is 0 uncertainty as to which pair is used. As such, the information cascade model does not predict any particular activity pattern for this condition (other than to predict that it involves contextual control); a more theoretically-motivated definition of task-set is required to make a more detailed prediction.

#### Levels of abstraction analyses

The levels of abstraction account classifies the conditions in terms of nesting level, rather than the quantity of information provided by each type of cue. This leads to a different grouping of task factors: the condition with 0 levels of nesting (i.e. baseline) should be isolated, all conditions with 1 level of nesting (i.e. all low-abstraction conditions) should be grouped together, and all conditions with 2 levels of nesting (i.e. all high-abstraction conditions) should be grouped together (see Figure 3). Reorganizing the conditions with this method isolates the multiple-trial conditions relative to the single-trial conditions as did the previous information theoretic analysis, but now, the maintenance duration contrast is not confounded with level of abstraction, and it is possible to identify whether any effects seen in the episodic control contrast in the information theoretic set of analyses relate to the duration of maintenance, or instead an increase in the level of abstraction. The resulting 2 (abstraction: low vs. high)×2 (maintenance duration: single- vs. multiple-trial) ANOVA was performed on the same ROIs identified by the blocked contrast above, because this analysis also predicts that the high abstraction, multiple-trial condition should elicit greater responses than baseline.

Additionally, one additional contrast (low abstraction - baseline, collapsing across the duration manipulation in the low abstraction conditions) was investigated via a paired t-test in order to investigate the prediction regarding the activity pattern across different areas lateral PFC. As stated above, the abstraction hypothesis makes two predictions regarding the functional organization of lateral PFC. First, mid-DLPFC should show increased activation in the high abstraction condition relative to both the low level of abstraction and the active baseline condition (i.e. it should demonstrate a main effect of the abstraction manipulation, but no difference between the low abstraction conditions and baseline). Second, posterior PFC should show an increased response in both the low and high abstraction conditions relative to baseline. These predictions indicate that both posterior PFC and mid-DLPFC should show increased responses in the high-abstraction case relative to baseline. In contrast, different responses for each region are predicted in the low-abstraction conditions: posterior PFC should be more responsive to the low-abstraction condition than mid-DLPFC. Because of this, a linear contrast investigating the parametric effect of abstraction would not be appropriate in discriminating between sub-regions of PFC that are sensitive to different levels of abstraction (a linear contrast across three conditions effectively measures the difference between the two most extreme conditions, and is insensitive to the response of the intermediate condition).

#### Decomposition of Temporal Dynamics

After investigating blocked effects, a more sophisticated first-level model was used to estimate parameter values for both transient activations associated with particular trial events (i.e. event-related effects) and for sustained activity associated with entire task blocks. Sustained effects can be independently coded into the GLM, using an assumption of a fixed-shape response of long duration (i.e., boxcar convolved with a gamma function). The logic of this approach is that the transient effects should be decaying back to baseline during inter-trial intervals, whereas sustained effects should remain relatively constant and of increased amplitude relative to blocks of fixation. Transient effects were analyzed by estimating values for the various time points within the hemodynamic response (i.e. using a Finite Impulse Response basis set). This approach to GLM coding of transient and sustained responses has been validated in both simulation and empirically based methodological studies [22]. The duration of the transient (event-related) epoch was taken to be 25 s (10 scanning frames). Event-related activation amplitude was calculated as the difference between the fourth and first time points of the hemodynamic epoch. Finally, the magnitude estimates for transient and sustained effects for each individual participant were submitted to a group analysis using random-effects model ANOVAs or t-tests.

The primary question of interest was whether the transient or sustained effects were consistent with the adaptive context maintenance hypothesis, and in particular, whether the temporal dynamics of individual brain areas changed as a function of task demands. The analyses relating the different temporal dynamics to the blocked effects involved submitting the transient and sustained amplitudes to a repeated measures ANOVA with level of abstraction (low vs. high), duration (multiple- vs. single trial), and activity dymanics (sustained vs. transient) as within-subjects factors.

### Terminology associated with different areas of PFC

Because different researchers use different terms to refer to different areas of PFC, we will be explicit in adopting the terminology used by Badre & D'Esposito [2]. We will use dorsal premotor cortex (PMd) to refer to those identified areas of PFC that are superior to a Z coordinate of 45. We will use posterior PFC to refer to those areas of PFC that are inferior to the PMd Z cutoff, and posterior to a Y coordinate of 14. We will use mid-dorsolateral PFC (mid-DLPFC) to refer to areas of PFC anterior to the posterior PFC cutoff, and posterior to a Y coordinate of 41. These coordinates were calculated in a data-driven approach by averaging the most extreme values from the bordering areas identified by either Badre & D'Esposito, 2007 or Koechlin et al, 2003 [2], [4]. For example, the Y cutoff marking the border of mid-dorsolateral PFC with posterior PFC was chosen by averaging the most anterior coordinate reported as posterior PFC in either study (Y = 10, [9]) with the most posterior coordinate reported as mid-DLPFC (Y = 18, also in [9]).

## Supporting Information

Analysis S1 **Investigation of sustained and transient activity associated with the information-cascade and levels-of-abstraction hypotheses.**
(PDF)
