# Supplementary material for: The Function and Organization of Lateral Prefrontal Cortex: A Test of Competing Hypotheses
Source: PLoS One. 2012 Feb 15;7(2):e30284. doi: 10.1371/journal.pone.0030284 (PMC3280249; doi:10.1371/journal.pone.0030284)
Supplement: Analysis S1 — Investigation of sustained and transient activity associated with the information-cascade and levels-of-abstraction hypotheses. (PDF) [file pone.0030284.s001.pdf]

The function and organization of lateral prefrontal cortex: A test of competing hypotheses: Analysis S1

Jeremy R. Reynolds[1,*], Randall C. O'Reilly[2], Jonathan D. Cohen[3] Todd S. Braver[4]
1 Department of Psychology, University of Denver, Denver, Colorado USA 80208
2 Department of Psychology, University of Colorado Boulder, Boulder, Colorado USA 80305
3 Department of Psychology and Princeton Neuroscience Institute, Princeton University, Princeton, New Jersey USA 08540
4 Department of Psychology, Washington University, St Louis, Missouri USA 30044
∗ E-mail: jeremy.reynolds@psy.du.edu

Analysis S1: Investigation of sustained and transient activity associated with the information cascade and levels-of-abstraction hypotheses.

The main text presents analyses of *blocked* activity associated with the information-cascade and levels-of-abstraction hypotheses. As the investigation of the adaptive context maintenance hypothesis illustrates, the current design allowed for the decomposition of these blocked responses into sustained and transient components. Because the information cascade and levels-of-abstraction hypotheses made no predictions regarding particular activation dynamics, the blocked contrasts were used to maximize statistical power. Nevertheless, the following sections present analyses of the decomposed sustained and transient responses.

Information Cascade Hypothesis

Analysis of the decomposed activity revealed that the blocked effects associated with the information-cascade hypothesis were carried by a combination of sustained and transient activity. Episodic control had an effect on only one ROI, via increased sustained activity [PMd; center of mass: -34, -7, 55; $F(1,28)=3.87$, $p=0.06$, $\eta_p^2 = 0.12$]. Contextual control had an effect on four ROIs, all via transient activity (both ROIs in mid-DLPFC, the ventral area of posterior-PFC, and the most posterior area of PMd) [min $F(1,28)=4.13$, $p=0.05$, $\eta_p^2 = 0.13$]

Levels of Abstraction Hypothesis

Analysis of the decomposed activity revealed that the blocked effects associated with the levels-of-abstraction hypothesis were carried by transient activity, as all areas demonstrating a blocked effect of abstraction (high vs. low) also demonstrated a corresponding transient increase [min $F(1,28)=3.13$, $p=0.09$, $\eta_p^2 = 0.10$]. Similarly, the areas demonstrating increased blocked activity in the low-abstraction conditions relative to baseline also demonstrated this effect in their transient activity [min $F(1,28)=3.16$, $p=0.09$, $\eta_p^2 = 0.10$].

Interestingly, Focusing on the transient activity made the dissociation between mid-DLPFC and posterior PFC stronger: These two ROIs demonstrated a significant ROI x level of abstraction interaction on transient activity [$F(1,28)=7.7$, $p=0.01$, $\eta_p^2 = 0.22$]. However, this interaction was the *opposite* of that predicted by the abstraction hypothesis. Mid-DLPFC demonstrated a larger difference between the low abstraction condition and baseline (mean difference = 0.15%) than did posterior PFC (mean difference =-0.01%).
